# A shared alarmone-GTP switch underlies triggered and spontaneous persistence

**DOI:** 10.21203/rs.3.rs-5679108/v1

**Published:** 2025-01-03

**Authors:** Jue Wang, Danny Fung, Jessica Barra, Jeremy Schroeder, Jin Yang, Fukang She, Megan Young, Daniel Amador-Noguez, David Ying

**Affiliations:** University of Wisconsin Madison; University of Wisconsin Madison; University of Wisconsin Madison; University of Michigan; University of Wisconsin-Madison; University of Wisconsin Madison; University of Wisconsin Madison; University of Wisconsin-Madison; University of Wisconsin Madison

## Abstract

Persisters describe phenotypically switched cells refractory to antibiotic killing in a genetically susceptible population, while preserving the ability to resume growth when antibiotics are discontinued1,2. Since its proposal 70 years ago, great strides were made to build the framework regarding persistence, including defining triggered, spontaneous and antibiotic-induced persisters. However, challenges remain in characterizing the molecular determinants underlying the phenotypic switch into persistence3. Here we document triggered, spontaneous and antibiotic-induced persistence in a Gram-positive bacterium, all through a common switch involving the alarmone (p)ppGpp and each stemming from a different alarmone synthesis pathway. Starvation-triggered persistence is mediated by Rel synthetase, and spontaneous persistence is through self-amplification via allosteric enzyme activation of alarmone synthetases Rel and SasB, whereas lethal and sublethal concentrations of cell wall antibiotics induce alarmones through an antibiotic-induced alarmone synthetase SasA, consequently enabling adaptive persistence that promotes survival. (p)ppGpp accumulation promotes persistence by depleting intracellular GTP and antagonizing its action. We developed a fluorescent GTP reporter to visualize rare events of persister formation in wild type bacteria, revealing a rapid switch from growth to dormancy in single cells as their GTP levels drop beneath a threshold. While a modest drop of GTP in bulk population slows down growth and promotes antibiotic tolerance, (p)ppGpp drives persistence by allowing the switch-like dynamics to drop GTP beneath the persister threshold in single cells. Persistence through alarmone-GTP antagonism is likely a widespread mechanism to survive antibiotics in Gram positive bacteria and possibly beyond.

## Main Text

Antibiotic persisters are individual bacterial cells that survive prolonged bactericidal antibiotic treatment despite being genetically sensitive to antibiotics^1–3^. Joseph Bigger originally proposed that persisters can be generated before or after antibiotics treatment^1^. Later, persisters were categorized by their pathways of generation-starvation-triggered, spontaneously generated and antibiotic-induced^3^-yet their molecular mechanisms remain poorly understood. Although persistence can be triggered by nutrient deprivations^4^ through intracellular accumulation of the alarmone (p)ppGpp^5–9^, it is also argued that that slow growth or a reduction in ATP levels, rather than (p)ppGpp, is responsible for triggering persistence^10,11^. In addition to starvation, persisters can form spontaneously^2^. However, spontaneous persisters exist in natural populations at low frequency, making their identification challenging and understanding the mechanisms of their formation even more difficult. While toxin-antitoxin (TA) modules are strongly implicated in persistence^2,5,8,12,13^, their roles in spontaneous antibiotic persistence of wild type bacteria remain unclear^14^. Finally, persisters were observed both before and after treatment with antibiotics^15^, and bacteriostatic antibiotics are known to induce persisters^16^. However, it is difficult to differentiate antibiotic-induced persistence from antibiotic tolerance^3^, spontaneous persistence, transcriptional response to specific drugs, or byproduct of cellular damage from drug treatment^3^. The mechanisms of antibiotic-induced persistence remain poorly understood.

**Figure 1:**
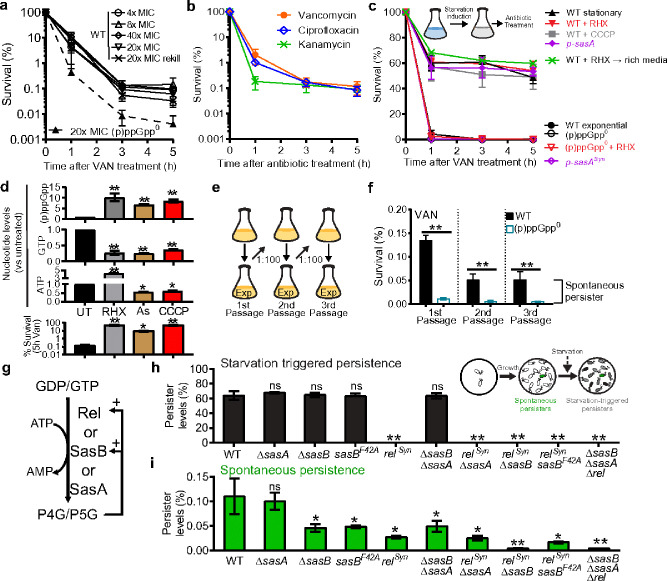
(p)ppGpp mediates starvation triggered and spontaneous persistence. (a) Survival curves of Bacillus subtilis wild type (WT) and (p)ppGpp-null mutant ((p)ppGpp0) to lethal concentrations of vancomycin (VAN). MIC: minimal inhibitory concentration. Rekill: cells which survived 20x MIC vancomycin treatments were regrown and treated with vancomycin again. (b) Survival curves of prophage-cured wild type to 20× MIC vancomycin, 20× MIC ciprofloxacin or 8x MIC kanamycin. (c) Starvation-trigggered persistence of WT depends on (p)ppGpp. Biphasic kill curves of vancomycin treatment were performed after several starvation or (p)ppGpp inducing conditions: stationary: stationary phase, RHX: pre-treated with amino acid starvation inducer arginine hydroxamate, +CCCP: pre-treated with ATP synthesis inhibitor CCCP, p-sasA or p-sasASyn: (p)ppGpp0 strains overexpressing the (p)ppGpp synthetase SasA or its synthetase-dead variant SasASyn (SasAD87G). See supplemental data fig 4 for plots in log scale. (d) Nucleotide levels in cells pre-treated with RHX, or ATP synthesis inhibitors CCCP or arsenate (As) for 30 min. Nucleotide levels were measured by thin layer chromatography (TLC) and normalized to their levels before induction. Bottom panel represents persistence to vancomycin treatment of the pre-treated cells. (e-f) Survival of serial-passaged populations to vancomycin for 5 h. Persisters which remained through the passage were defined as spontaneous persisters3. (g) In B. subtilis and many gram-positive bacteria, (p)ppGpp is synthesized by three (p)ppGpp synthetases. Rel is the bifunctional (p)ppGpp synthetase and hydrolase, while SasB (SAS1/RelQ/YjbM) and SasA (SAS2/RelP/YwaC) are monofunctional synthetases. (h) Persister levels in starvation-induced (black) WT and (p)ppGpp mutants with RHX. (i) Levels of spontaneous persisters (green, 2nd passage) in WT and (p)ppGpp mutants. relSyn (RelD264G) is a synthetase inactive variant of Rel which retained its hydrolase activity essential for viability in the presence of sasB and sasA. sasBF42A encodes an allosteric pppGpp binding site mutant of SasB. Values represent mean (n >= 3) and error bars indicate s.d. *: p < 0.05, **: p < 0.01, ns: not significant (Student’s t test).

Here we directly visualize the formation of starvation-triggered, spontaneous and antibiotic-induced persisters in the Gram-positive model bacterium *Bacillus subtilis* and reveal that multiple cellular mechanisms of persistence can converge on a common pathway of alarmone-GTP antagonism. To quantify persistence in *B. subtilis*, we began by treating cells with the cell wall-targeting antibiotic vancomycin at concentrations 20 times higher than its minimal inhibitory concentration (Fig 1a and Supplemental Data Fig 1). The results revealed biphasic killing kinetics, with a rapid elimination of the majority of cells followed by the prolonged survival of a small subpopulation. This surviving fraction (~0.1%) remained consistent across varying drug concentrations and, upon regrowth, became re-sensitized to antibiotics (Fig 1a), confirming that these are phenotypically switched persisters rather than genetically altered resisters^1,3,17^. We also tested the effect of prophages on persistence, given that persistence against DNA-damaging antibiotic ciprofloxacin has been shown to be complicated by prophage activation in *E. coli*^14^. We confirmed that, similar to *E. coli*, persistence against ciprofloxacin is weakened by prophages (Supplemental Data Fig 2), but persistence against vancomycin remains unaffected regardless of the presence or absence of resident prophages SPβ and PBSX within the genome. Furthermore, the levels of persisters in prophage-cured cells were consistent across different classes of antibiotics, including vancomycin, ciprofloxacin, and kanamycin, which targets translation (Fig 1b). This suggests that the persisters originate from a common subpopulation that is refractory to multiple antibiotics^3^.

(p)ppGpp is a starvation-induced alarmone strongly implicated in antibiotic survival^9^. We therefore examined the effect of (p)ppGpp on antibiotic resistance, tolerance and persistence using a (p)ppGpp^0^ mutant, which lacks all three (p)ppGpp synthetases^18^. Compared to the wild type, the (p)ppGpp^0^ strain exhibited similar resistance to a variety of antibiotics (MIC assay, Supplemental Data Fig 1), a modestly reduced antibiotic tolerance (~5% reduction of MDK_99_, Supplemental Data Fig 3a-b), and strongly reduced antibiotic persistence (~10 fold down, Fig 1a, Supplemental Data Fig 3c). These results indicate that (p)ppGpp predominantly promotes antibiotic persistence.

Antibiotic persistence can be triggered by starvation or occur spontaneously^3^. To investigate the role of (p)ppGpp in starvation-triggered persistence, we starved cells using several methods: allowing growth into stationary phase, inducing amino acid starvation, or depleting ATP with arsenate or CCCP. We then perform kill curves with vancomycin under these conditions or after starvation is removed by back-diluting in rich media. The biphasic kill curves (Fig 1c, Supplemental Data Fig 4a) showed a high level of triggered persisters (~500-fold increase from 0.1% to ~50%) upon all starvation treatments and even after starvation-removal, consistent with the established definition of triggered persistence^3^.

Interestingly, all starvation conditions also induced (p)ppGpp accumulation. We observed (p)ppGpp buildup not only upon amino acid starvation but also during ATP depletion (Fig 1d, Supplemental Data Fig 5). Furthermore, starvation-triggered persistence was abolished in the (p)ppGpp^0^ mutant, indicating that (p)ppGpp induction is necessary for triggered persistence (Supplemental Data Fig 4b-c). Finally, we found that even in the absence of starvation, gratuitous induction of (p)ppGpp by overexpressing its synthetase^19^ was sufficient to produce high levels of persisters (Fig 1c, *p-sasA*). These findings demonstrate that (p)ppGpp accumulation is both necessary and sufficient to trigger persistence.

We then investigated the occurrence of spontaneous persistence. To differentiate spontaneous persisters from those triggered by starvation, we employed a classical serial passage experiment^2,3^ (Fig 1e). In this method, cells are repeatedly diluted into fresh medium during exponential growth, which removes starvation-triggered persisters from the lag phase culture while leaving a consistent, basal fraction of spontaneously generated persisters. This approach enables us to identify a rare population of spontaneous persisters (~0.05%) after the starvation-trigerred persisters have been eliminated (Fig 1f). Notably, the spontaneous persisters were absent in the (p)ppGpp^0^ mutant, indicating that (p)ppGpp plays a key role in both spontaneous and starvation-triggered persistence.

In *B. subtilis*, (p)ppGpp is synthesized by three enzymes: Rel, SasA, and SasB (Fig 1g). To assess their roles in persister formation, we analyzed single, double, and triple mutants of these synthetases (Fig 1h–i, Supplemental Data Fig 6, 7). Our findings show that only the starvation-responsive synthetase, Rel, is essential for starvation-triggered persistence (Fig 1h). In contrast, spontaneous persistence is controlled by both Rel and SasB, as mutations in either *rel* or *sasB* significantly reduce spontaneous persister levels, and in combination, they completely abolish this phenotype (Fig 1i). Rel can be activated by pppGpp (Supplemental Data Fig 8), aligning with recent findings^20^. SasB, a tetrameric enzyme, enables allosteric activation of (p)ppGpp synthesis by pppGpp, with a Hill coefficient of ~3.0^21^. A mutation that disrupts the allosteric pppGpp-binding site in SasB (*sasB*^*F42A*^) leads to a similar reduction in spontaneous persistence as observed in the Δ*sasB* mutant (Fig 1i). These results suggest that self-amplification of (p)ppGpp synthesis is crucial for spontaneous persister formation.

**Figure 2:**
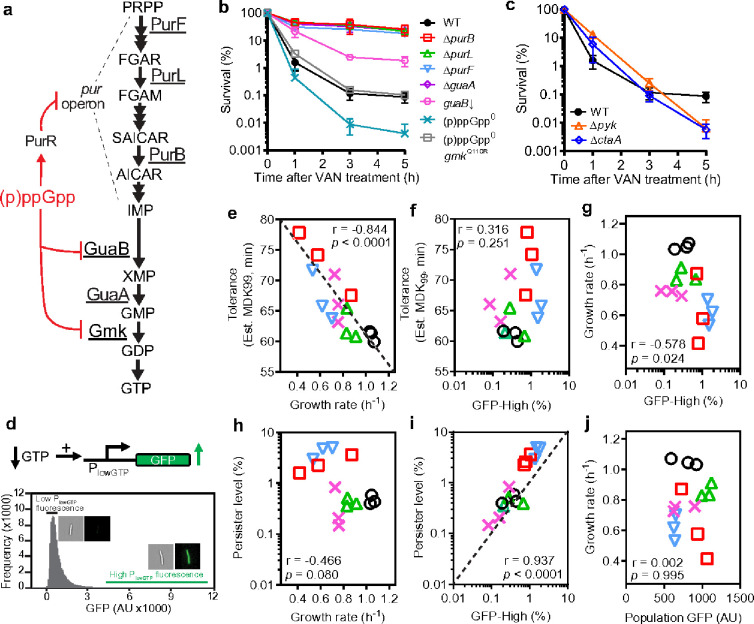
(p)ppGpp mediates persistence by reducing GTP and is independent of tolerance. (a) (p)ppGpp robustly inhibits de novo GTP biosynthesis by inhibiting the GDP synthetase Gmk and the IMP dehydrogenase GuaB, as well as inhibiting expression of the pur operon through the PurR repressor. (p)ppGpp also inhibits GTP synthesis by purine salvage which is not illustrated here. (b-c) Vancomycin survival curves of wild type (WT), purine biosynthesis mutants ΔpurB, ΔpurL, ΔpurF, GTP synthesis mutants ΔguaA and guaB repressed mutant (guaB↓), slow growth mutants Δpyk (pyruvate kinase) and ΔctaA (heme synthase), as well as (p)ppGpp and (p)ppGpp gmkQ110R. (d) Measurement of low GTP cells in bacterial population. The PlowGTP reporter is activated when cellular GTP level is low. Flow cytometry analysis of wild-type population containing the reporter (~1.5×106 cells). Representative image of cells with low or high PlowGTP fluorescence was shown. (e-j) Correlation between growth rate, tolerance, pesistence, population-averaged PlowGTP signals, and frequency of low GTP cells (high PlowGTP fluorescence cells) in wild-type under different growth conditions. Wild type containing the PlowGTP reporter were grown in minimal media with different carbon sources. Populations were measured for their growth rate, PlowGTP fluorescence, and levels of high PlowGTP fluorescence cells using flow cytometry (~1.5×106 cells), as well as tolerance and persistence from vancomycin survival curves as shown in Supplemental Data Fig 21. Each value represents a single replicate. Three replicates for each condition were shown. r, Pearson correlation coefficient, p, p-value.

Next, we search for the downstream effector of (p)ppGpp that mediates antibiotic persistence. (p)ppGpp is a bacterial alarmone with pleiotropic functions including upregulating competence and sporulation^22^. We found that disruption of competence, sporulation, or three known TA systems in *B. subtilis* has little impact on persistence (Supplemental Data Fig 9). (p)ppGpp also serves to antagonize the essential nucleotide GTP in *B. subtilis* and to downregulate macromolecular biosynthesis^18^. (p)ppGpp production consumes GTP, and (p)ppGpp directly inhibits multiple enzymes along the pathway of GTP production (Fig 2a)^23–27^. In addition, (p)ppGpp binds to the purine transcription regulator PurR to inhibit transcription of the *pur* operon, encoding *de novo* purine biosynthesis genes upstream of GTP synthesis^28^. Furthermore, (p)ppGpp competes with GTP to regulate macromolecule synthesis enzymes including the DNA replication enzyme primase^29,30^ and GTPases for ribosome biogenesis, assembly and protein translation^31–34^. In starvation conditions that trigger persisters, GTP levels were significantly depleted, whereas ATP levels vary (Fig 1d), suggesting that GTP depletion may be a common effector leading to persistence.

To test whether GTP depletion mediates antibiotic persistence even in the absence of (p)ppGpp induction, we used our previously published inducible strain that downregulated the expression of the guanine nucleotide synthesis gene *guaB*^35^, which lowered GTP without inducing (p)ppGpp (Supplemental Data Fig 10). We observed an increased persistence by ~10-fold (*guaB*↓, Fig 2b), indicating that GTP depletion is sufficient to generate persisters. In addition, we performed a non-biased Tn-Seq analysis in *B. subtilis* to identify genes whose disruption increased cell survival after prolonged antibiotic treatment (Supplemental Data Fig 11). The strongest hits mapped to 12 *de novo* purine biosynthesis genes in the *pur* operon and *guaA* encoding GMP synthetase (Supplemental Data Fig 11b, c, Supplemental Data Table 5–6). We verified that disruption of the *pur* operon or *guaA* reduces purine levels including GTP (Supplemental Data Fig 11d), with corresponding kill curves confirming increased antibiotic persistence (Fig 2b–c, Supplemental Data Fig 11e). Next, we wanted to know whether reducing GTP in complete absence of (p)ppGpp can still promote persistence. Through genetic screen, we identified a mutation in *gmk* encoding guanylate kinase (see Methods) that reduces GTP levels of the (p)ppGpp^0^ strain back to the wild type level (Supplemental Data Fig 12). This mutant [(p)ppGpp^0^ *gmk*^Q110R^] restored persistence back to wild type (Fig 2b–c, Supplemental Data Fig 12), confirming that GTP depletion is sufficient for antibiotic persistence.

In addition to increased persistence, we also noted a modest rise in antibiotic tolerance in the GTP synthesis mutants (Supplemental Data Fig 7), which aligns with our previous findings that GTP depletion leads to slower growth^35^. Slower growth has been shown to contribute to antibiotic tolerance^11,36^. This raises the question: Is increased antibiotic persistence simply a consequence of slow growth, and thus always correlated with tolerance?

Genetic screens in *Bacillus subtilis* have identified slow-growing mutants, some of which deplete GTP while others do not. We tested a couple of slow-growing mutants that do not rely on GTP depletion. Intriguingly, while these mutants exhibited increased tolerance, they do not show an increase in persistence; in fact, some showed decreased persistence (Fig 2c). This suggests that, although tolerance and persistence are correlated in certain mutants or conditions^11^, they can be genetically uncoupled.

To further distinguish between tolerance and persistence, we grew wild-type cells in different carbon sources, measuring their growth rates and antibiotic killing dynamics (Supplemental Data Fig 21a-b). Our results revealed a strong correlation between slower growth rates and increased tolerance (Fig. 2e), but no significant correlation between growth rates and persistence (Fig. 2h). This demonstrates that, while tolerance and persistence can share overlapping characteristics, they represent distinct biological phenomena that can be independently regulated.

Our findings, while unexpected, are explainable: although both slow growth and antibiotic tolerance are population-level phenomena, persistence reflects the behavior of a phenotypically heterogeneous subpopulation of cells functioning as outliers. To differentiate population-based tolerance from the phenotypically heterogeneous subpopulation of persisters, it is critical to measure GTP levels in single cells.

To achieve this, we constructed a fluorescent reporter for GTP levels in single cells. Building on our prior result that the *ilvB* gene is induced by ~20-fold upon GTP depletion via the CodY repressor-which uses GTP as a co-repressor^37,38^-we cloned the CodY operator from *ilvB* to control fluorescent protein expression ectopically (Supplemental Data Fig 13a). This reporter, termed P_lowGTP_ (Figure 2d), is fully repressed in (p)ppGpp^0^ cells, with fluorescence indistinguishable from background autofluorescence (Supplemental Data Fig 13b). In (p)ppGpp+ cells, the reporter displays a dose-dependent increase in fluorescence across a physiological range of (p)ppGpp induction and GTP depletion (Supplemental Data Fig 13c, 13d). This response to amino acid or ATP starvation is abolished in (p)ppGpp^0^ mutants (Supplemental Data Fig 14). Additionally, neither the P_lowGTP_ reporter nor the CodY regulator interferes with antibiotic survival (Supplemental Data Fig 15). The P_lowGTP_ reporter activity is inversely related to the ribosomal P1 promoter activity (Supplemental Data Fig 16), which is activated by GTP^35^. Although increased fluorescence could theoretically result from a failure to dilute the fluorescent protein during slow growth, our data show that P_lowGTP_ fluorescence respond to changes in GTP levels rather than slow growth (Supplemental Data Fig 17).

After calibrating the P_lowGTP_ reporter in bulk population, we examined whether it reflects GTP levels in individual cells. FACS sorting of cells with high P_lowGTP_ fluorescence, followed by LC-MS analysis, confirmed that the bright cells had significantly lower GTP levels (Supplemental Data Fig 18). This demonstrates that the reporter accurately reflects GTP levels at both the population and single-cell levels. Together, these results confirm that our reporter reliably tracks GTP depletion rather than slow growth, enabling us to distinguish between slow-growing, tolerant cells and GTP-depleted persister subpopulations.

Using this reporter, we examined GTP distributions in single cells with flow cytometry (Fig 2d, Supplemental Data Fig 19). Most cells are dark during growth in rich medium, consistent with bulk measurements (Supplemental Data Fig 13b). However, we identified a heavy-tailed subpopulation of rare, bright cells that depended on the presence of the reporter (Fig. 2d, Supplemental Data Fig 19), indicating significant GTP depletion. This subpopulation was absent in (p)ppGpp^0^ cells but reappeared in (p)ppGpp^0^*gmk* cells with rescued persistence (Supplemental Data Fig 19b).

The fractions of bright, low-GTP cells varied when cells were grown in media with different carbon sources (Supplemental Data Fig 21c, d). Strikingly, the fraction of low-GTP cells strongly correlated with the fraction of persisters measured via kill curves (Fig 2i, Supplemental Data Fig 20b, 5-hour survivors), but not correlated with growth rate (Fig 2g) or antibiotics tolerance (Fig 2f, Supplemental Data Fig 21d). These results distinguish persistence from tolerance, demonstrating that persistence under various growth conditions is driven by the fraction of low GTP outliers, not by the population’s average growth rate.

**Figure 3:**
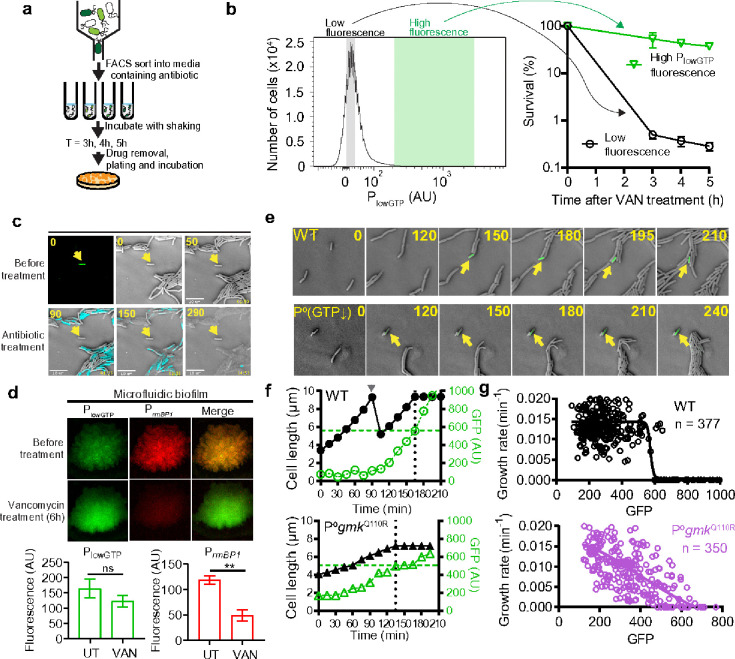
Direct observation of persistence switch in cells through (p)ppGpp-GTP antagonism. (a) Schematic of FACS sorting and antibiotic survival assay using the PlowGTP reporter. Bacterial population containing the PlowGTP reporter were sorted based on PlowGTP fluorescence and immediately treated with antibiotic. Survival was measured by serial dilution and plating followed by colony counting. (b) Wild type containing PlowGTP-GFP reporter was FACS-sorted into low or high PlowGTP fluorescence. Approximately 1 × 106 cells were sorted per sample per replicate. Fractions were treated with vancomycin and measured for survival over time. Values represent mean ± s.d., n = 3. See also supplemental data figure 22. (c) Time lapse micrograph of wild type cells containing the PlowGTP reporter in response to antibiotic treatment. Exponentially growing wild type containing the PlowGTP reporter were patched on agarose pads made with growth media. High PlowGTP fluorescence cells were identified (arrow) and monitored for antibiotic survival using carbenicillin and Sytox-blue staining (cyan) for viability. Values in yellow indicate time in min. (d) Survival of low GTP cells in biofilm. Wild type containing both PlowGTP (for low GTP, green) and PrrnBP1 (for high GTP, red) reporters were grown into colony biofilm followed by vancomycin treatment for 6 h. Bar graphs indicate the changes in high GTP and low GTP populations before and after treatment. Four random regions in the biofilm were measured. Values represent mean (n >= 3) and error bars indicate s.d. *: p < 0.05, **: p < 0.01, ns: not significant (Student’s t test). (e) Representative single cell dynamic trace of persister formation. Cells were patched onto agarose pad made with growth media at roughly one cell per field of view, and the monitored for growth till before saturation (see also supplemental data figure 24a-b). A total of roughly 30,000 cell growth and division events were recorded (~150 events per microcolony) to reveal ~10 persistence entrance events. Numbers indicate time in min. Arrows indicate a cell with increased PlowGTP fluorescence followed by growth attenuation. (f) Quantitated changes in cell length vs PlowGTP fluorescence (left) vs PlowGTP fluorescence (right) from the single trace in (e). Dotted lines indicate persistence entrance. (g) Summary data of specific growth rate and reporter fluorescence from ~100 independent single cell traces similar to e (ten entrance traces). Solid lines indicate non-linear regression (variable slope) model fitted to the data.

Despite the strong correlation between persisters and low-GTP cells, it is essential to determine if these two subpopulations were indeed the same. To explore this, we used two methods. First, we applied FACS to sort exponentially growing wild-type cells containing the P_lowGTP_ reporter (Fig 3a). FACS-isolated cells with high P_lowGTP_ fluorescence (top 0.1%) were immediately treated with vancomycin. After 5 hours of treatment and subsequent antibiotic removal, cells were plated. ~80% of the sorted low-GTP cells formed colonies compared to only ~0.1% survival in the low-fluorescence population (Fig 3b). This demonstrates that nearly all low-GTP cells are antibiotic-refractory persisters capable of regrowth after antibiotic removal. We confirmed that this persistence was specifically dependent on P_lowGTP_, using a differentially labeled P_veg_ control that measured the effect of protein dilution (Fig 3b, Supplemental Data Fig 22), or through fluorescent protein swapping (Supplemental Data Fig 22).

In addition to FACS sorting, we employed time-lapse microscopy to monitor cell fate during antibiotic treatment. We found that only bright cells were non-growing before antibiotic treatment and remained viable during the treatment, while the rest of the cells, which elongated and divided, was rapidly killed (see Fig 3c, Supplementary Video 1 for an example).

To further investigate persisters in biofilms, where they are commonly found^39^, we developed a microfluidic biofilm system with confocal microscopy (Supplemental Data Fig 23). Using dual labeled cells (P_*rrnBP1*_ and P_lowGTP_), we identified a mixture of persisters and growing cells in microfluidic biofilms (Fig 3d). Upon prolonged (6 hour) antibiotic treatment, the low-GTP cells survived within the biofilm (Fig 3d), confirming that the majority of persisters in biofilms are low-GTP cells.

After confirming that P_lowGTP_-bright cells are persisters, we investigated how these persisters form before antibiotic treatment. Our goal was to observe how rare, spontaneous persisters emerge from actively growing wild-type cells, and to examine how this process relates to GTP levels. We seeded highly diluted, growing cells on solid growth media and monitored ~30,000 growth and division events (Supplemental Data Fig 24a-b). From this, we identified ten cells that entered a persister state before reaching high cell density (Fig 3e, Supplemental Data Fig 24c-k, Supplementary Video 2). We retrospectively analyzed the dynamics of their transition into persistence by quantifying their growth, division, and GTP reporter activity. This revealed distinct, switch-like behaviors at the single-cell level (Fig 3f, Supplemental Data Fig 24c-k). Initially, each pre-persister cell maintained the same elongation rate as actively growing cells, while its P_lowGTP_ fluorescence gradually increased, often over more than one cell division (Supplemental Data Fig 24). This was followed by a sudden stop in elongation, marking entry into dormancy when the P_lowGTP_ fluorescence reached a threshold (Fig 3f, Supplemental Data Fig 25). In all cases, the reporter fluorescence rose before growth inhibition occurred (Fig 3f, Supplemental Data Fig 25), indicating that GTP depletion triggers growth inhibition rather than resulting from it.

The fluorescence thresholds were remarkably consistent across different persister entry events (Fig 3g), suggesting that the switch to persistence is activated when intracellular GTP levels fall to a specific threshold. This threshold aligns with values seen in our microscopy and flow cytometry experiments, corresponding to an estimated GTP concentration of ~0.1–0.2 mM^40^. This range is comparable to what is observed in wild-type cells experiencing strong (p)ppGpp-accumulation (Supplemental Data Fig 13c-d), where most cells became starvation-triggered persisters.

While GTP depletion leads to persistence, what is the role of (p)ppGpp in persister dynamics? Interestingly, the (p)ppGpp^0^ *gmk*^Q110R^ mutant, which has GTP levels similar to wild-type cells but lacks (p)ppGpp (Fig 2c), can still form spontaneous persisters (Fig 3f, Supplemental Data Fig 25j-l, Supplemental Video 3). However, these mutants do not exhibit the switch-like dynamics characteristic of persister entry in wild-type cells. Instead, their entry into dormancy is gradual, with elongation slowing as reporter activity increases (Fig 3f). Moreover, in these mutants, GTP levels and growth rates show a linear relationship (Fig. 3g), contrasting with the switch-like relationship seen in wild-type cells. Thus, (p)ppGpp promotes GTP depletion and enabling the rapid, switch-like entry into persistence seen in wild-type cells.

**Figure 4:**
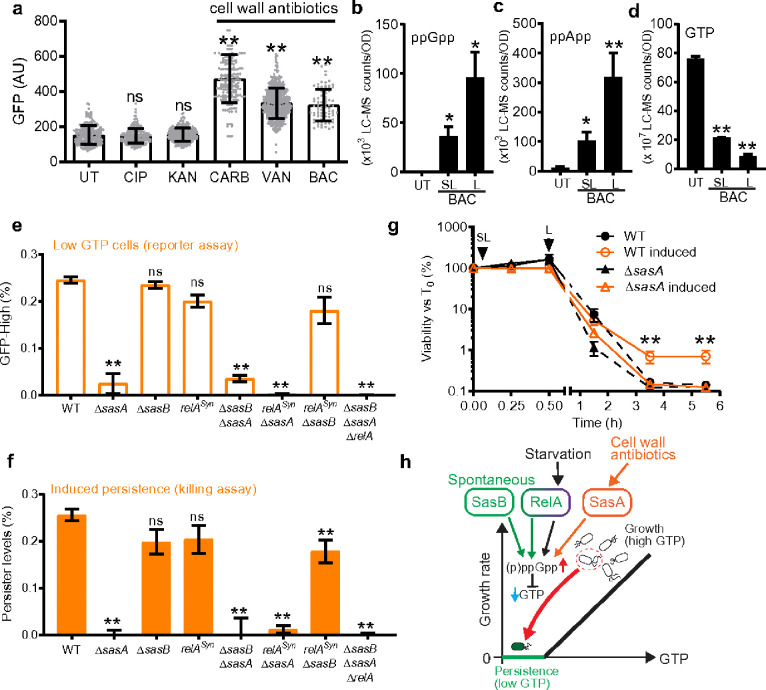
Antibiotic-induced persistence through alarmone accumulation contributes to adaptive survival. (a) Cell wall antibiotics induce PlowGTP reporter fluorescence. WT containing the PlowGTP reporter were treated with 20× MIC ciprofloxacin (CIP), 4× MIC kanamycin (KAN), 200× MIC carbenicillin (CARB), 20x MIC vancomycin (VAN) or 3× MIC bacitracin (BAC) for 2 h. Fluorescence was measured using microscopy (> 200 cells each, n = 3) UT: before treatment. (b to d) Levels of ppGpp, ppApp and GTP in wild type (WT) treated with sublethal or lethal concentrations of bacitracin for 0.5 h. UT: before treatment, SL: 0.5× MIC bacitracin, L: 3x MIC bacitracin. (e-f) Levels of cell wall antibiotic-induced persisters in WT and (p)ppGpp mutants. (e) Strains containing the PlowGTP reporter before and after 0.5× MIC bacitracin treatment for 0.5 h were measured for generation of low GTP cells using flow cytometry (~1×106 cells per sample per replicate, n = 3, see also Supplemental data figure 28). Levels of induced low GTP cells were determined by subtracting the levels in uninduced populations. (f) Survival of WT or (p)ppGpp mutants pretreated with 0.5× MIC for 0.5 h, followed by lethal (3 × MIC) bacitracin treatment for up to 5 h. Levels of induced persistence were determined by subtracting survival of uninduced populations at 5 h. See also Supplemental data figure 29. Values represent mean and error bars represent s.d. Statistical tests were done between WT and mutant pairs. *: p < 0.05, **: p < 0.01, ns: not significant (Student’s t test). (g) Survival curve of WT or ΔsasA pretreated with sublethal (SL, 0.5× MIC) bacitracin for 0.5 h, followed by lethal (L, 3 × MIC) bacitracin treatment for up to 5 h. Statistical tests were done between WT and mutant pairs. *: p < 0.05, **: p < 0.01, ns: not significant (Student’s t test). (h) Model for persistence pathways mediated by (p)ppGpp-GTP antagonism. Starvation-triggered persistence (purple) is mediated primarily through the (p)ppGpp synthetase Rel. Spontaneous persistence is mediated through activities of two (p)ppGpp synthetases Rel and SasB (green). Persisters can also be induced by cell wall antibiotics through SasA (orange). Thus, (p)ppGpp enables integration of different signals to trigger persistence through GTP depletion.

Finally, we used our newly established system to investigate the poorly understood phenomenon of antibiotic-induced persistence^3^. We found that P_lowGTP_ fluorescence increased in cells exposed to lethal concentrations of cell wall antibiotics, including carbenicillin, vancomycin and bacitracin (Fig 4a, Supplemental Data Fig 27a). While most cells were killed by irreversible damage, a few P_lowGTP_-bright cells survived and resumed growth after the antibiotics were removed (Supplemental Data Fig 27b-c, Supplementary Video 4). LC-MS analysis revealed that exposure to both lethal and sublethal doses of bacitracin led to the accumulation of alarmones, such as ppGpp and ppApp, and a decrease in GTP levels (Fig 4b–d). These results suggest that cell wall antibiotic treatment induces alarmone production and depletes GTP. Using mutants of (p)ppGpp synthetases, we found that a single alarmone synthetase, SasA^19^, is responsible for (p)ppGpp accumulation and GTP depletion during cell wall antibiotic treatment (Fig 4e, Supplemental Data Fig 28).

To assess whether this alarmone response provides a protective effect, we measured persistence due to treatment with sublethal concentrations (below MIC) of bacitracin (Fig 4f, 4g Supplemental Data Fig 29). While sublethal bacitracin did not impact cell viability, it did induce SasA expression (Supplemental Data Fig 30)^41,42^. Remarkably, when followed by a lethal dose of bacitracin (3x MIC), cells pretreated with sublethal antibiotics showed approximately five times more persisters than non-pretreated cells, with minimal differences in tolerance (Fig 4g). This survival mechanism, dependent on SasA but not other alarmone synthetases (Fig 4f), allows pre-exposed cells to better resist lethal antibiotic exposure. Therefore, antibiotic-induced persistence may serve as an adaptive strategy, significantly enhancing cell survival against a later lethal antibiotic challenge.

Our data highlight the pivotal role of (p)ppGpp in persistence. We demonstrate that conditions triggering persistence^10^ also induce (p)ppGpp accumulation, that persistence depends on (p)ppGpp accumulation, and that three (p)ppGpp synthetases are differentially responsible for starvation-triggered, spontaneous and antibiotic-induced persistence (Fig 4h). While (p)ppGpp senses environmental signals to initiate persistence, GTP antagonism serves as the critical effector, likely via coordinated suspension of multiple GTP-dependent processes such as protein translation and DNA synthesis (Supplemental Data Fig 31). Our work also distinguishes persisters from tolerators, revealing that persisters arise through specific genetic pathways that generate individual cells with low GTP, independent of population-wide growth rates or bulk GTP levels.

We propose a new model for spontaneous persistence. Phenotypic switch into persistence can be mediated by competition between toxins and antitoxins in TA modules to provide a threshold-like mechanism^2,12,43^. Our findings suggest that cells can also utilize an alternative mechanism for the spontaneous entrance: through allosteric enzyme cooperativity and self-amplification of (p)ppGpp synthesis (Supplemental Data Fig 32). This enables rapid amplification of a small signal, such as from noise in (p)ppGpp synthesis, to allow spontaneous persister formation in rare individual cells.

Our study also provides strong evidence for bactericidal antibiotic-induced persistence. While previous research suggested that antibiotics induce persistence through cellular damage^15^ and stress responses such as the SOS pathway^44^, our results show that antibiotics, particularly cell wall-targeting ones, induce persistence via the accumulation of alarmones like (p)ppGpp, driven by SasA expression in response to cell envelope damage^41,45,46^. In the soil bacterium *B. subtilis*, the evolution of this regulation may be shaped by a fitness advantage from its habitat to enhance survival to diffusible antimicrobials from neighboring microbes. For example, bacitracin, which we show to induce persistence, is a diffusible natural antibiotic produced by other soil *Bacillus* species^47^. Clinically, this model explains why (p)ppGpp mutants in pathogens such as *S. aureus* show reduced survival against cell wall-targeting drugs^48^ and diminished clearance under combination antibiotic therapies^49^. Thus, the alarmone-dependent persistence mechanism is likely conserved across various bacterial species.

Finally, persistence may play a key role in the emergence of genetic resistance by expanding the reservoir of surviving bacteria during antibiotic treatment. Given that antibiotic overuse increases bacterial exposure to these drugs, driving the evolution of resistance^50^, targeting persister formation could improve treatment outcomes and help curb the rise of antibiotic-resistant infections.

## Methods

### Bacterial strains and strain construction

All bacterial strains, plasmids and oligonucleotides used in this study are listed in Supplemental Data Table 1 to 4. LB and LB agar were used for cloning and maintenance of strains. For selection in *B. subtilis*, media were supplemented with the following antibiotics when required: spectinomycin (80 μg/mL), chloramphenicol (5 μg/mL), kanamycin (10 μg/mL), and tetracycline (10 μg/mL). The combination of lincomycin (12.5 μg/mL) and erythromycin (0.5 μg/mL) was used to select for MLS resistance. Carbenicillin (100 μg/mL) was used for selection in *E. coli*.

*B. subtilis* (p)ppGpp biosynthesis mutants were constructed by transformations of integration plasmids containing an I-sceI endonuclease cut site and regions of homology upstream and downstream of specific synthetase genes (pJW300 for Δ*sasB*, pJW370 for *sasB*^F42A^, pJW306 for Δ*sasA*, and pJW371 for *rel*^D264G^) followed by transformation of pSS4332 for marker-less recombination^51^. Successful recombination was verified by PCR. For the construction of (p)ppGpp^0^ mutant, Δ*sasA* Δ*sasB* was transformed with Δ*rel*::*mls* PCR product from genomic DNA using oligos oJW902/oJW903 followed by MLS resistance selection^25^. Construction of integration plasmid for *sasB*^F42A^ was done by site-directed mutagenesis of pJW370 by PCR using oligos oJW2309/oJW2310.

The (p)ppGpp^0^ *gmk*^Q110R^ mutant was obtained from isolating suppressor mutants from (p)ppGpp^0^ cells by plating on S7 minimal medium plates containing 1% glucose. The surviving colonies were plated on S7 minimal medium plates containing 0.5% casamino acids and 0.5 mM 8-azaguanine, or S7 minimal medium plates containing 0.5% casamino acids and 0.1 mM guanosine^25^ to differentiate between mutants containing mutation in HprT or Gmk. Colonies which can grow on guanosine but not 8-azaguanine were sequenced to identify the mutant *gmk* allele. Whole genome sequencing was performed to confirm that *gmk*^Q110R^ is the only mutation in the strain.

The *guaB*^down^ (*guaB*↓) mutant in (p)ppGpp+ background was constructed by transformation of pJW305^25^, which replaces the chromosomal copy of *guaB* with a IPTG-inducible copy of *guaB* (P_*spac*_-*guaB*). This allows controllable *guaB* expression using IPTG during strain construction and growth to avoid generation of suppressors.

*B. subtilis* deletion mutants were constructed by serial transformation of PCR products from the *B. subtilis* knockout collection (BGSC, Gross lab)^52^. Where required, the lox-site flanked *erm*^R^ or *kan*^R^ cassette was removed using pDR244-*cre* followed by selection for the loss of MLS or Kan resistance.

Construction of P_lowGTP_ fluorescence reporters was done by fusion of PCR products containing the P_lowGTP_ promoter (primers oJW1935/oJW1936) with coding regions of fluorescence proteins (primers oJW1995/oJW1996 for GFP or oJW2805/oJW2806 for mCherry) using ligase cycling reaction (LCR)^53^. The promoter-fluorescence protein gene fusions were cloned into pDR110 backbone flanked by *amyE* without the P_*spank*_ promoter for subsequent transformation.

For the construction of P_*rrnBP1*_-GFPns (unstable GFP sequence from Griffith *et al.*^54^) reporter, DNA fragments of P_*rrnBP1*_ (primers oJW2083/oJW2084), GFPns (primers oJW1995/oJW2020), flanking regions of *lacA* (primers oJW1990/oJW2414 and oJW2413/oJW2082), and lox-site flanked *erm*^R^ cassette (primers oJW2133/oJW2134) were amplified by PCR using synthetic oligonucleotides or genome DNA. The resulting PCR products were fused by LCR followed by amplification using PCR to generate linear recombination fragment of *lacA*::P_*rrnBP1*_-GFPns-lox-*ermR*-lox for transformation.

For the construction of P_*sasA*_-mCh reporter, DNA fragments of P_*sasA*_ (primers oJW3099/oJW3079), mCh (primers oJW2805/oJW2806), flanking regions of *lacA* (primers oJW1990/oJW2414 and oJW2413/oJW2082), and lox-site flanked *erm*^R^ cassette (primers oJW2133/oJW2134) were amplified by PCR using synthetic oligonucleotides or genome DNA. The resulting PCR products were fused by LCR followed by amplification using PCR to generate linear recombination fragment of *lacA*::P_*sasA*_-mCh-lox-*ermR*-lox for transformation.

For the construction of P_veg_-GFP reporter, DNA fragments of P_veg_ (primers oJW3928/oJW2806), GFP (primers oJW1995/oJW1996), flanking regions of *lacA* (primers oJW1990/oJW2414 and oJW2413/oJW2082), and lox-site flanked *erm*^R^ cassette (primers oJW2133/oJW2134) were amplified by PCR using synthetic oligonucleotides or genome DNA. The resulting PCR products were fused by LCR followed by amplification using PCR to generate linear recombination fragment of *lacA*:: P_veg_-GFP-lox-*ermR*-lox for transformation.

Removal of the lox-site flanked *erm*^R^ cassette was done by transformation with pDR244-*cre* and selected for the loss of MLS resistance. All mutants and constructs were verified by DNA sequencing.

### Growth conditions

*Bacillus subtilis* strains were grown in S7 defined medium^55^; MOPS was used at 50 mM rather than 100 mM, supplemented with 0.1% glutamate, 1% glucose, and 0.5% casamino acids. Growth of YB886 strain background was supplemented with 20 μg/mL tryptophan and 50 μg/mL methionine. For growth in minimal media, both glutamate and casamino acids were replaced with 200 μg/mL L-isoleucine, 200 μg/mL L-leucine, and 200 μg/mL L-valine, and 1% carbon sources were used as indicated. Routinely, cells from young colonies on overnight LB-agar plates at 37 °C (< 12 h) were inoculated into growth media and then grown at 37 °C with 250 rpm shaking. Cultures in logarithmic phase (OD600 ≈ 0.1–0.3) were treated with antibiotics or inducers, including arginine hydroxamate (RHX, 0.5 mg/mL), carbonyl cyanide m-chlorophenyl hydrazone (CCCP, 5 μM), sodium azide (NaN_3_, 4 mM), or arsenate (2.5 mM). Isopropyl β-D-1-thiogalactopyranoside (IPTG) was added to a final concentration of 0.5 mM to induce *guaB* expression from an IPTG-inducible promoter (P_*spac*_), while depletion of *guaB* expression was done by omitting IPTG in the growth media.

The following inducers and concentrations were used unless otherwise specified: RHX: 0.5 mg/mL; CCCP: 5 μM; NaN_3_: 4 mM; Arsenate: 2.5 mM; CARB: 0.5 μg/mL (0.5x MIC) or 100 μg/mL (200x MIC); BAC: 64 μg/mL (0.5x MIC) or 384 μg/mL (3x MIC); CIP: 0.1 μg/mL (0.5x MIC) or 4 μg/mL (20x MIC); KAN: 0.625 μg/mL (0.3x MIC) or 8 μg/mL (8x MIC); VAN: 0.1 μg/mL (0.5x MIC) or 4 μg/mL (20x MIC)) along with non-induction controls.

### Minimum inhibitory concentration (MIC) determination

MICs for chloramphenicol (CAM), tetracycline (TET), kanamycin (KAN), ciprofloxacin (CIP), norfloxacin (NOR), rifampicin (RIF), bacitracin (BAC) and vancomycin (VAN) were determined using the microdilution method^56^. Logarithmic phase cells were back-diluted to a final titer of ≈ 5×10^5^ CFU/mL into 96-well plates containing two-fold serial dilutions of respective antibiotics in S7_50_ medium with 0.1% glutamate, 1% glucose, and 0.5% casamino acids. After 16–20 hours of incubation at 37 °C with 250 rpm shaking, the MIC was determined as the lowest drug concentration that prevented visible growth.

### Bacterial growth measurement

For growth measurement, fresh colonies of *Bacillus subtilis* strains on LB agar were resuspended into different growth media as specified and diluted to OD600 ≈ 0.005 in 96-well plates. Growth was monitored by OD600 at 37°C under shaking in the Synergy2 microplate reader (BioTek). Doubling times were estimated by fitting the growth data to the exponential growth curve using a custom python script.

### Persister assay

To prepare exponentially growing *B. subtilis* populations, cells from young colonies on overnight LB-agar plates at 37 °C (< 12 h) were inoculated into S7_50_ medium with 0.1% glutamate, 1% glucose and 0.5% casamino acids and grown to OD600 ≈ 0.1–0.3 at 37°C, 250 rpm. For growth in minimal medium, both glutamate and casamino acids were replaced with 200 μg/mL L-isoleucine, 200 μg/mL L-leucine, and 200 μg/mL L-valine, and 1% carbon sources were used as indicated. Treatments with bactericidal antibiotics were done at the following concentrations: CIP, 4 μg/mL (20× MIC); VAN, 4 μg/mL (20× MIC), KAN, 8 μg/mL (8× MIC), BAC, 384 μg/mL (3× MIC). To determine cell viability, culture aliquots were taken at T=0 and at designated times after treatment, serially diluted, and plated onto Luria-Broth (LB) agar. Plates were incubated at 37°C overnight. Viability at different time points was determined as CFU/mL and relative survival (vs T_0_) was calculated.

For experiments involving pre-induction of cells with (p)ppGpp-inducing agents, cells were grown to OD600 ≈ 0.1 and divided into two cultures: one containing the inducing agent (RHX, 0.5 mg/mL; BAC, 64 μg/mL; CCCP, 5 μM; NaN_3_, 4 mM; arsenate, 12.5 mM) and other as non-induction control. The cultures were grown for an additional 30 min under the same conditions (T=0.5 hr) and subjected to the persister assay, as described above.

In the case of measuring spontaneous persistence, we followed the definition of spontaneous persisters that they are generated during growth in a non-stressed condition^2,3^. To achieve this, early exponentially growing cultures were 1:100 back-diluted into fresh media and regrew to exponential phase (OD600 ≈ 0.1–0.3) for one or two rounds followed by antibiotic treatment^3^.

### Estimation of antibiotic tolerance

For the estimation of population tolerance, we adopted the definition of minimum duration of killing for 99% of the population (MDK_99_)^3^. To exclude the contribution of persisters to population tolerance, we determined the level of persisters in the biphasic killing curve and subtracted this subpopulation from the bulk population. MDK_99_ of the population was estimated from the logarithmic killing phase in the killing curve.

### Measurement of intracellular nucleotides by thin-layer chromatography

To measure intracellular nucleotides, cells were first harvested from overnight plates, back-diluted to OD_600_ = 0.005, and grown in low-phosphate (0.1X phosphate, 0.5 mM) S7_50_ medium with 0.1% glutamate, 1% glucose, and 0.5% casamino acids. Once cultures reached OD_600_ ≈ 0.05, 1 mL cells were labeled with 50 μCi of ^32^P orthophosphate (900 mCi/mmol; Perkin Elmer) for 2–3 generations before treatment or sampling. At OD_600_ ≈ 0.15, RHX, CCCP, or arsenate were added to the cultures and samples were collected at regular time points for nucleotide extraction. Nucleotides were extracted by incubating 100 μL cells with 20 μL of 2 M formic acid on ice for at least 20 minutes. Samples were spotted on PEI cellulose thin-layer chromatography (TLC) plates (Selecto) and resolved in 1.5 M or 0.85 M potassium phosphate monobasic (KH_2_PO_4_, pH 3.4) buffer to separate (p)ppGpp or GTP, respectively. TLC plates were exposed on storage phosphor screens (GE Healthcare) and scanned on a Typhoon imager (GE Healthcare).

### Measurement of intracellular nucleotides by LC-MS

LC-MS quantification of nucleotides was performed as described previously^19^. Cells were grown in S7_50_ medium supplemented with 20 amino acids^19^ to OD_600_ ≈ 0.3 at 37°C, 250 rpm before harvesting. 25 mL of cultures were sampled and filtered through PTFE membrane (Sartorius). For experiments involving bacitracin induction, cells were harvested before and after 0.25× MIC (sublethal) or 1.25× MIC (lethal) bacitracin treatment for 30 min. Membranes with cell pellet were submerged in 3 mL extraction solvent mix (on ice 50:50 (v/v) chloroform/water) to quench metabolism, lyse the cells and extract metabolites. Mixtures of cell extracts were centrifuged at 5000× g for 10 min to remove organic phase, and then centrifuged at 20000× g for 10 min to remove cell debris. Samples were analyzed using HPLC-MS system consisting of a Vanquish UHPLC system linked to electrospray ionization (ESI, negative mode) to a Q Exactive Orbitrap mass spectrometer (Thermo Scientific) operated in full-scan mode to detect targeted metabolites based on their accurate masses. LC was performed on an Acquity UPLC BEH C18 column (1.7 μm, 2.1 × 100 mm; Waters). Total run time was 30 min with a flow rate of 0.2 mL/min, using Solvent A as denoted above and acetonitrile as Solvent B. The gradient was as follows: 0 min, 5% Solvent B; 2.5 min, 5% Solvent B; 19 min, 100% Solvent B; 23.5 min 100% Solvent B; 24 min, 5% Solvent B; 30 min, 5% Solvent B. Quantification of metabolites was performed by using MAVEN software^57^ and normalized to OD_600_ at the time of cell harvest.

For LC-MS analysis of FACS-sorted cells, membranes with filtered cells (about 8 × 10^6^ cells) were submerged in 1.5 mL extraction solvent mix (methanol:acetonitrile:H2O=40:40:20) to quench metabolism, lyse the cells and extract metabolites. The cell extract was centrifuged at 21,000 × g for 10 min at 4°C, and 1 mL of supernatant was transferred to a new microcentrifuge tube and dried completely with SpeedVac. Dried metabolites were then resuspended in 100 μL Solvent A (97:3 (v/v) water/methanol, 10 mM tributylamine pH~8.2–8.5 adjusted with ~9 mM acetic acid). Samples were analyzed using an HPLC-tandem MS (HPLC-MS/MS) system consisting of a Vanquish UHPLC system linked to heated electrospray ionization (ESI, negative mode) to a hybrid quadrupole high resolution mass spectrometer (Q-Exactive orbitrap, Thermo Scientific) operated in full-scan selected ion monitoring (MS-SIM) mode to detect targeted metabolites based on their accurate masses. MS parameters were set to a resolution of 140,000, an automatic gain control (AGC) of 3e6, a maximum injection time of 100 ms, and a scan range of 400–1000 mz. Only ions between 10–15 minutes retention time were scanned by MS. LC was performed on an Acquity UPLC BEH C18 column (1.7 μm, 2.1 × 100 mm; Waters). Total run time was 30 min with a flow rate of 0.2 mL/min, using Solvent A and 100% acetonitrile as Solvent B. The gradient was as follows: 0 min, 5% B; 2.5 min, 5% B; 19 min, 100% B; 23.5 min 100% B; 24 min, 5% B; and 30 min, 5% B. Raw output data from the MS was converted to mzXML format using in-house-developed software. Quantification of metabolites was performed by using MAVEN software^57^ and were normalized to an internal standard of six most represented nucleotides detected in the sample.

### Fluorescence microscopy

To monitor (p)ppGpp induction using fluorescence reporters, cells were grown to OD600 ≈ 0.1–0.3 followed by 30-min induction with the following inducers at concentrations listed below unless otherwise specified: RHX: 0.5 mg/mL; CCCP: 5 μM; NaN_3_: 4 mM; Arsenate: 2.5 mM; CARB: 0.5 μg/mL (0.5× MIC) or 100 μg/mL (200× MIC); BAC: 64 μg/mL (0.5× MIC) or 384 μg/mL (3× MIC); CIP: 0.1 μg/mL (0.5× MIC) or 4 μg/mL (20× MIC); KAN: 0.625 μg/mL (0.3× MIC) or 8 μg/mL (4× MIC); VAN: 0.1 μg/mL (0.5× MIC) or 4 μg/mL (20× MIC)), along with non-induction controls.

All imaging samples were spotted on 1.5% agarose pads made with the same growth medium, and immediately imaged with Olympus IX-83 inverted microscope (Olympus) using 60X phase contrast objective with fluorescence filters (excitation: 470/20 nm, dichroic mirror: 485 nm, emission: 515/50 nm for GFP; excitation: 575/20 nm, dichroic mirror: 595 nm, emission: 645/90 nm for mCherry or propidium iodide; and excitation: 427/10 nm, dichroic mirror: 595 nm, emission: 472/30 nm for Sytox blue). Single-cell time-lapse imaging was performed at 15-min intervals for each field at 37°C using temperature-controlled imaging chamber (Tokai Hit) coupled with automatic stage and microscope control as described previously^58^. The measurement was generally over the course from the birth of the cell until the time lapse stopped due to crowding of the microcolony or in rare cases severe drifting of focus. When comparing phenotypes between strains, both strains were imaged in parallel on the same imaging dish using the same microscope with same settings. For imaging persister survival in time-lapse experiments, final concentrations of 5 μg/mL CARB (10x MIC) and 200 nM Sytox blue or propidium iodide (Molecular Probes) were applied to the agarose pads at designated times. To remove CARB after treatment, 5 units/mL final concentration of penicillinase (Sigma) was applied. Strains without the fluorescence reporters were used for autofluorescence measurement.

### Biofilm growth and imaging

*B. subtilis* biofilms were grown on a custom microfluidic device fabricated with polydimethylsiloxane (PDMS). The device contains a central chamber connected to inlet and outlet media channels which allows constant media flow through the central chamber. A semi-permeable dialysis membrane was fixed on top of the central chamber to provide a platform for biofilm growth. This setup allows diffusion of nutrients or small molecules from the media flow underneath to support biofilm growth on the membrane and allow subsequent treatment with antibiotics. To grow *B. subtilis* biofilms, 1 μL of early exponential phase culture (OD_600_ ≈ 0.05) was applied on the membrane and grown at room temperature (25°C) for 24 h under constant flow of S7_50_ medium supplemented with 0.5% glutamate and 0.5% glycerol.

Imaging of biofilms was performed using IXplore SpinSR confocal imaging microscope (Olympus) using 20X phase contrast objective with fluorescence filters (488 nm laser with 510–550 nm emission filter for GFP; 561 nm laser with 575–625 nm emission filter for mCherry). Biofilms at 24h post-inoculation were imaged before and after switching to the same growth medium containing 4 μg/mL Vancomycin. 51 stacks at 1 μm intervals were taken for each time point. Images were projected along z-axis from the top of the biofilm using maximum intensity projection using the cellSense software (Olympus). For quantitation of reporter signals within the biofilm, we sampled ~4 random regions within the center of the biofilm and measured their GFP and mCherry intensities. Background fluorescence was measured from regions without biofilm and used for background subtraction.

### Flow cytometry and cell sorting

Flow cytometry was performed at UWCCC flow cytometry core. To prepare samples for flow cytometry, cells from young colonies on overnight LB-agar plates at 37 °C (< 12 h) were inoculated into and grown in S7_50_ medium with 0.1% glutamate, 1% glucose and 0.5% casamino acids to OD_600_ ≈ 0.1–0.3 at 37°C, 250 rpm. For growth in minimal medium, both glutamate and casamino acids were omitted and 1% carbon sources were used as indicated. Cells were immediately fixed with 0.4% paraformaldehyde for 15 min at room temperature, washed 3 times with 1× phosphate buffered saline (PBS), and kept at 4°C until analysis. Fixation was verified by viability plating and microscopy. Flow cytometry analysis was performed using BD LSRFortessa flow cytometer (BD Biosciences) with a 70-μm nozzle. Cell populations were detected using both forward and side scatter (FSC and SSC). Single-cell fluorescence was measured using 488 nm laser and detection filters for GFP (530/30 nm, 505LP dichroic filter). Autofluorescence was measured by analyzing parental strains without the fluorescence reporter and subtracted from the raw reporter fluorescence. Approximately 1.5 million events were measured for each sample. For the determination of antibiotic induced persistence, the frequency of low GTP cells after antibiotic induction was subtracted from the frequencies before induction.

FACS was performed at UWCCC flow cytometry core. To prepare samples for cell sorting, cells were harvested from young colonies on overnight LB-agar plates at 37 °C (< 12 h) and grew in S7_50_ medium with 0.1% glutamate, 1% glucose and 0.5% casamino acids to OD_600_ ≈ 0.3 at 37°C, 250 rpm. FACS analysis was performed using BD FACSAria cell sorter (BD Biosciences) with a 70-μm nozzle at room temperature using 488 nm laser and 530/30 nm detection filters for GFP, and 561 nm laser and 610/20 nm detection filters for mCherry. Auto-fluorescent cells were eliminated by gating with isogenic strain without the fluorescent reporters. At least 1,000 cells were obtained from the rarest gate for each sample. Cell recovery rate was estimated to be > 90% based on viability counting on LB plates. For antibiotic treatment, cells were directly sorted in to tubes containing 4x MIC of VAN followed by shaking at 37 °C. Aliquots were taken at different times for serial dilution and plating to measure survival by colony counting. Number of cells before treatment (T_0_) were measured by the cell sorter.

For FACS-sorting of cells for LC-MS analysis, cells containing the P_lowGTP_ reporter were grown in S7_50_ medium with 0.1% glutamate, 1% glucose and 0.5% casamino acids to either exponential (OD600 ≈ 0.2) or stationary phase (OD_600_ ≈ 4.0). Both populations were mixed in 10:1 ratio, and immediately FACS-sorted into low or high fluorescence fractions. Auto-fluorescent cells were eliminated by gating with isogenic strain without the fluorescent reporters. Approximately 8 × 10^6^ cells were sorted and filtered on PTFE membrane (Sartorius) to remove the sheath fluid. Membranes with filtered cells were then subjected to metabolite extraction and LC-MS analysis. CFU counts were performed from a small aliquot of the sorted fractions to account for potential variations in the number of cells sorted between fractions.

### Transposon sequencing (Tn-Seq)

*B. subtilis* 168 transposon mutant library was kindly provided by the Grossman Lab^59^. Construction of the library is briefly described as follows: *In vitro* transposition of *B. subtilis* 168 genomic DNA (gDNA) with magellen6× transposon was performed by mixing 1.3 μg pCJ41 (containing magellen6x transposon), 34 ng purified MarC9 transposase, 5 μg *B. subtilis* gDNA, 10 μL 2x buffer A (41 mM HEPES pH 7.9, 19% glycerol, 187 mM NaCl, 19 mM MgCl2, 476 μg/mL BSA and 3.8 mM DTT) into 20 μL reaction *in vitro* and incubated overnight at 30°C. The transposed DNA was precipitated and resuspended in 2 μL 10x buffer B (500 mM Tris-Cl pH 7.8, 100 mM MgCl_2_, 10 mM DTT), 2 μL 1 mg/mL BSA and 11 μL H_2_O followed by 4h incubation at 37°C. After incubation, 4 μL 2.5 mM dNTPs and 1 μL of 3U/μL T4 DNA Polymerase were added to the DNA and further incubated for 20 min at 12°C, followed by heat inactivation at 75°C for 15 min. Next, 0.2 μL 2.6 mM NAD and 1 μL of 10 U/μL *E. coli* DNA ligase were added and the reaction was incubated at 16°C overnight. The resulting in vitro transposed and repaired gDNA was transformed into *B. subtilis* 168 and plated on LB agar containing spectinomycin and incubated overnight. Colonies containing the transposon were washed off and pooled into a single library. The library was estimated to contain ~50,000 unique transposon inserts across the genome.

For the selection experiment with the transposon library, an aliquot of the library was inoculated and grown in S7_50_ medium with 0.1% glutamate, 1% glucose and 0.5% casamino acids supplemented with tryptophan (20 μg/mL) at 37°C, 250 rpm. At OD600 ≈ 0.1–0.3, cultures were treated with 20x MIC vancomycin or ciprofloxacin. Cultures before and after antibiotic treatment were plated onto LB plates and recovered after ~14 h incubation at 37°C. ~650,000 colonies from each sample were pooled and snap-frozen for genomic DNA extraction and sequencing library preparation.

Preparation of the sequencing library was performed as previously described^60^. Frozen cell pellets were resuspended in 500 μL lysis buffer with lysozyme and RNase A (20 mM Tris-HCl pH 7.5, 50 mM EDTA, 100 mM NaCl, 2 mg/mL lysozyme, 120 μg/mL RNase A) and incubated at 37°C for 20–30 min. Next, the incubated cell lysate was mixed with 60 μL 10% N-lauroylsarkosine and further incubated at 37°C for 15 min. Genomic DNA (gDNA) was purified using 600 μL phenol, then 600 μL phenol:chloroform:isoamyl alcohol (25:24:1), and finally 600 μL pure chloroform. DNA in the aqueous phase was precipitated using 1/10 volumes of 3M NaOAc and 2 volumes of 100% ethanol. DNA pellet was washed with 70% ethanol, air-dried on bench, and resuspended in 10 mM Tris-HCl, pH 8.5 and stored at 4°C. For each sample, 6 μg of DNA was used for MmeI digestion in 200 μL (6 μg gDNA, 6 μL MmeI (2000 U/mL, NEB), 0.5 μL 32 mM S-adenosylmethionine, 20 μL NEB CutSmart Buffer, and ddH_2_O up to 200 μL). DNA was digested for 2.5 h at 37°C, after which 2 μL calf intestinal phosphatase (10,000 U/mL, NEB) was added to the digest and incubated for 1h at 37°C. Digested gDNA was extracted with 200 μL phenol:chloroform:isoamyl alcohol (25:24:1) followed by 200 μL of pure chloroform. DNA in the aqueous phase was first mixed with 1/10 volume of 3M NaOAc and 67 ng/mL glycogen, and then 2.5 volumes of 100% ethanol. The tubes were then placed at −80°C for 20 min and then centrifuged at max speed for 15 min at 4°C. Precipitated DNA was washed with 150 μL 70% ethanol twice at room temperature, air-dried, and resuspended in 15 μL ddH2O.

For annealing of the DNA adaptor, 20 μL of 100 μM synthesized oligos (IDT) were mixed with 1 μL of 41 mM Tris-HCl pH 8.0 (final concentration of adaptor: 50 μM in 1mM Tris-HCl pH 8.0). Oligos were annealed by heat denaturation (95 °C for 5 min) and stepwise cool-down (94°C for 45 s then repeat with −0.3°C per cycle for 250 cycles, then hold at 15°C) using PCR machine. Annealed adaptors were diluted to 3.3 μM in ddH2O and stored at −20°C.

For adaptor ligation, 5 μL of digested DNA were mixed with 1 μL of 3.3 μM DNA adaptor, 1 μL of 10× T4 DNA ligase buffer (NEB), 1 μL of T4 DNA ligase (400,000U/mL, NEB) and 2 μL ddH_2_O. The ligation mix was incubated overnight at 16°C in a PCR machine.

Amplification of adaptor-ligated DNA library was performed using barcoded primers and Phusion high-fidelity DNA polymerase (NEB) for 18 cycles according to provided instructions. The PCR products were mixed in equal amounts, purified by size-exclusion, and submitted for sequencing using Illumina sequencing primer (5’-ACACTCTTTCCCTACACGACGCTCTTCCGATCT-3’). Deep sequencing was done using Illumina HiSeq 2500 (Illumina) by the University of Michigan DNA Sequencing Core. Analysis of sequencing data was done using a custom Python script and mapped to the *B. subtilis* 168 reference genome (NC_000964.3). Visual inspection of transposon insertion profiles was done using GenomeBrowse (Golden Helix).

### Expression and purification of B. subtilis Rel

The plasmid for Rel purification was constructed as follows. The *B. subtilis rel* coding sequence was PCR amplified from NCIB3610 genomic DNA using primers oJW3196/oJW3197. The pE-SUMO expression vector was amplified using primers oJW3194/oJW3195. The PCR products were assembled to generate pJW753 by Golden Gate assembly (New England BioLabs). Plasmids were verified by DNA sequencing.

To express His_6_-SUMO-Rel, fresh transformants of *E. coli* BL21 carrying pE-SUMO-*rel* were grown in LB at 37°C to OD600 ≈ 0.5, followed by 1:50 dilution into Terrific Broth and grown at 30°C until OD600 ≈ 1.5. His_6_-SUMO-Rel expression was induced with 1 mM IPTG for 4 h at 30°C. Cells were pelleted and stored at −80 °C until use. Frozen cell pellets were thawed on ice, resuspended in ice-cold lysis buffer (50 mM Tris-HCl, pH 8, 1M NaCl, 10 mM imidazole, DNase and cOmplete protease inhibitor (Roche)), and lysed with French press at 4°C. The cell lysate was centrifuged at 4°C, 13,000 rpm for 30 min to obtain the supernatant. Filtered supernatant was injected into an ӒKTA FPLC system (GE Healthcare) and passed through a HisTrap FF column (GE Healthcare). His_6_-SUMO-Rel was eluted with a gradient of Buffer A (50 mM Tris-HCl, pH 8, 1M NaCl, 5% glycerol, 10 mM imidazole) and Buffer C (50 mM Tris-HCl, pH 8, 1M NaCl, 5% glycerol, 500 mM imidazole). Fractions containing the protein were pooled with 300 μL SUMO protease into Spectra/Por dialysis tubing (Spectrum), and dialyzed into 50 mM Tris-HCl, 1M NaCl, 1mM β-mercaptoethanol and 5% glycerol overnight. Rel without the His_6_-SUMO tag was passed through the HisTrap FF column and then size-exclusion purified using a Superose 12 10/300 GL column (GE Healthcare) with ӒKTA FPLC system. Fractions containing the Rel protein were pooled and measured for its concentration by the Bradford Assay (Bio-rad). Aliquots were snap-frozen using liquid nitrogen and stored at −80 °C.

### In vitro pppGpp synthesis assay

*In vitro* pppGpp synthesis by Rel was monitored by measuring synthesis of radiolabeled pppGpp over time. The reaction contained 236 nM *B. subtilis* Rel, 0.05 μM [α^32^P] GTP, 1 mM ATP, 50 mM NaCl and 10 mM ATP in 20 mM Tris-HCl pH 7.5, with or without 10 μM non-radioactive pppGpp. The reaction was devoid of manganese to avoid potential effect of pppGpp hydrolysis. The reaction was initiated by addition of ATP and incubated at 37 °C. At indicated times, 10 μL of the reaction were mixed with 2 μL of 2M formic acid and chilled on ice for 20 min to quench the reaction. 1 μL samples were spotted on PEI cellulose thin-layer chromatography (TLC) plates (Millipore) and resolved in 1.5 M potassium phosphate monobasic (KH_2_PO_4_, pH 3.4) buffer to separate pppGpp. TLC plates were dried and exposed on storage phosphor screens (GE Healthcare) and scanned on a Typhoon imager (GE Healthcare).

### Quantification and Statistical Analysis

For TLC experiments, intensities of nucleotide spots were quantified using ImageQuant software (Molecular Dynamics). The raw intensities were corrected to the number of phosphates in the corresponding nucleotide and normalized to OD_600_ or ATP level before treatment (ATP_T=0_) for comparison between samples. For *in vitro* pppGpp synthesis assays, changes in pppGpp levels were normalized to T=0.

Microscopy image analysis and cell parameters (cell area and fluorescence intensity) measurements were done using Metamorph software (Molecular Devices). Background and autofluorescence were subtracted by comparing images obtained from identical strains without the fluorescence reporter. Single cell specific growth rate (μ) at each frame was calculated using the equation: $\mu =\frac{1}{Al}\frac{\Delta l}{\Delta t}$, where l is the cell length, Δl is the change in cell length and Δt is the change in time (in min). Flow-cytometry data were analyzed using FlowJo X software (FlowJo, LLC). Cells within a narrow range of cell sizes were gated, sub-gated to filter cell aggregates, and then measured for their fluorescence distribution.

Autofluorescence was measured using isogenic strains without the fluorescent reporters and subtracted from raw reporter signals. Gating for P_lowGTP_-high cells was set at 5-fold or higher above mean population fluorescence. This cut-off agrees with our FACS sorting experiment where cells with reporter fluorescence above this threshold are predominantly persisters. For the determination of antibiotic induced persistence, the frequency of low GTP cells after antibiotic induction was subtracted from the frequencies before induction.

Statistical information of individual experiments is included in the figure legends. n represents the number of biological replicates or number of cells for experiments involving single-cell measurements as indicated in the legends. Significance was tested using Student’s *t* test. Prism 7 (GraphPad) was used for statistical analysis.

## References

[1] Bigger J. Treatment of Staphylococcal Infections with Penicillin by Intermittent Sterilisation. The Lancet 244, 497–500, doi:10.1016/s0140-6736(00)74210-3 (1944).

[2] Balaban N. Q., Merrin J., Chait R., Kowalik L. & Leibler S. Bacterial persistence as a phenotypic switch. Science 305, 1622–1625, doi:10.1126/science.1099390 (2004). 15308767

[3] Balaban N. Q. Definitions and guidelines for research on antibiotic persistence. Nat Rev Microbiol, doi:10.1038/s41579-019-0196-3 (2019). PMC7136161 30980069

[4] Fung D. K., Chan E. W., Chin M. L. & Chan R. C. Delineation of a bacterial starvation stress response network which can mediate antibiotic tolerance development. Antimicrob Agents Chemother 54, 1082–1093, doi:10.1128/AAC.01218-09 (2010). 20086164 PMC2825962

[5] Kaspy I. HipA-mediated antibiotic persistence via phosphorylation of the glutamyl-tRNA-synthetase. Nat Commun 4, 3001, doi:10.1038/ncomms4001 (2013). 24343429

[6] Nguyen D. Active starvation responses mediate antibiotic tolerance in biofilms and nutrient-limited bacteria. Science 344, 982–986, doi:10.1126/science.1211037 (2011). PMC4046891 22096200

[7] Korch S. B., Henderson T. A. & Hill T. M. Characterization of the hipA7 allele of Escherichia coli and evidence that high persistence is governed by (p)ppGpp synthesis. Mol Microbiol 50, 1199–1213 (2003). 14622409 10.1046/j.1365-2958.2003.03779.x

[8] Helaine S. Internalization of Salmonella by macrophages induces formation of nonreplicating persisters. Science 343, 204–208, doi:10.1126/science.1244705 (2014). 24408438 PMC6485627

[9] Pacios O. (p)ppGpp and Its Role in Bacterial Persistence: New Challenges. Antimicrob Agents Chemother 64, doi:10.1128/AAC.01283-20 (2020). PMC7508602 32718971

[10] Conlon B. P. Persister formation in Staphylococcus aureus is associated with ATP depletion. Nat Microbiol, 16051, doi:10.1038/nmicrobiol.2016.51 (2016). PMC4932909 27572649

[11] Pontes M. H. & Groisman E. A. Slow growth determines nonheritable antibiotic resistance in Salmonella enterica. Sci Signal 12, doi:10.1126/scisignal.aax3938 (2019). PMC7206539 31363068

[12] Rotem E. Regulation of phenotypic variability by a threshold-based mechanism underlies bacterial persistence. Proc Natl Acad Sci U S A 107, 12541–12546, doi:10.1073/pnas.1004333107 (2010). 20616060 PMC2906590

[13] Moyed H. S. & Bertrand K. P. hipA, a newly recognized gene of Escherichia coli K-12 that affects frequency of persistence after inhibition of murein synthesis. J Bacteriol,, 768–775 (1983). 6348026 10.1128/jb.155.2.768-775.1983 PMC217749

[14] Harms A., Fino C., Sorensen M. A., Semsey S. & Gerdes K. Prophages and Growth Dynamics Confound Experimental Results with Antibiotic-Tolerant Persister Cells. MBio 8, doi:10.1128/mBio.01964-17 (2017). PMC5727415 29233898

[15] Johnson P. J. & Levin B. R. Pharmacodynamics, population dynamics, and the evolution of persistence in Staphylococcus aureus. PLoS Genet 9, e1003123, doi:10.1371/journal.pgen.1003123 (2013). 23300474 PMC3536638

[16] Westfall C. The widely used antimicrobial triclosan induces high levels of antibiotic tolerance in vitro and reduces antibiotic efficacy up to 100-fold in vivo. Antimicrob Agents Chemother, doi:10.1128/AAC.02312-18 (2019). PMC6496070 30782996

[17] El-Halfawy O. M. & Valvano M. A. Antimicrobial heteroresistance: an emerging field in need of clarity. Clin Microbiol Rev 28, 191–207, doi:10.1128/CMR.00058-14 (2015). 25567227 PMC4284305

[18] Anderson B. W., Fung D. K. & Wang J. D. Regulatory Themes and Variations by the Stress-Signaling Nucleotide Alarmones (p)ppGpp in Bacteria. Annu Rev Genet, doi:10.1146/annurev-genet-021821-025827 (2021). PMC12582393 34416118

[19] Fung D. K., Yang J., Stevenson D. M., Amador-Noguez D. & Wang J. D. Small Alarmone Synthetase SasA Expression Leads to Concomitant Accumulation of pGpp, ppApp, and AppppA in Bacillus subtilis. Front Microbiol 11, 2083, doi:10.3389/fmicb.2020.02083 (2020). 32983059 PMC7492591

[20] Roghanian M. (p)ppGpp controls stringent factors by exploiting antagonistic allosteric coupling between catalytic domains. Mol Cell 81, 3310–3322 e3316, doi:10.1016/j.molcel.2021.07.026 (2021). 34416138

[21] Steinchen W. Catalytic mechanism and allosteric regulation of an oligomeric (p)ppGpp synthetase by an alarmone. Proc Natl Acad Sci U S A 112, 13348–13353, doi:10.1073/pnas.1505271112 (2015). 26460002 PMC4629338

[22] Potrykus K. & Cashel M. (p)ppGpp: still magical? Annu Rev Microbiol 62, 35–51, doi:10.1146/annurev.micro.62.081307.162903 (2008). 18454629

[23] Wang B., Grant R. A. & Laub M. T. ppGpp Coordinates Nucleotide and Amino-Acid Synthesis in E. coli During Starvation. Mol Cell 80, 29–42 e10, doi:10.1016/j.molcel.2020.08.005 (2020). 32857952 PMC8362273

[24] Anderson B. W., Hao A., Satyshur K. A., Keck J. L. & Wang J. D. Molecular Mechanism of Regulation of the Purine Salvage Enzyme XPRT by the Alarmones pppGpp, ppGpp, and pGpp. J Mol Biol 432, 4108–4126, doi:10.1016/j.jmb.2020.05.013 (2020). 32446804 PMC7323586

[25] Kriel A. Direct regulation of GTP homeostasis by (p)ppGpp: a critical component of viability and stress resistance. Mol Cell 48, 231–241, doi:10.1016/j.molcel.2012.08.009 (2012). 22981860 PMC3483369

[26] Wang B. Affinity-based capture and identification of protein effectors of the growth regulator ppGpp. Nat Chem Biol 15, 141–150, doi:10.1038/s41589-018-0183-4 (2019). 30559427 PMC6366861

[27] Anderson B. W., Fung D. K. & Wang J. D. Regulatory Themes and Variations by the Stress-Signaling Nucleotide Alarmones (p)ppGpp in Bacteria. Annu Rev Genet 55, 115–133, doi:10.1146/annurevgenet-021821-025827 (2021). 34416118 PMC12582393

[28] Anderson B. W. The nucleotide messenger (p)ppGpp is an anti-inducer of the purine synthesis transcription regulator PurR in Bacillus. Nucleic Acids Res, doi:10.1093/nar/gkab1281 (2021). PMC8789054 34967415

[29] Wang J. D., Sanders G. M. & Grossman A. D. Nutritional control of elongation of DNA replication by (p)ppGpp. Cell 128, 865–875, doi:10.1016/j.cell.2006.12.043 (2007). 17350574 PMC1850998

[30] Giramma C. N., DeFoer M. B. & Wang J. D. The Alarmone (p)ppGpp Regulates Primer Extension by Bacterial Primase. J Mol Biol 433, 167189, doi:10.1016/j.jmb.2021.167189 (2021). 34389317 PMC8453095

[31] Corrigan R. M., Bellows L. E., Wood A. & Gründling A. ppGpp negatively impacts ribosome assembly affecting growth and antimicrobial tolerance in Gram-positive bacteria. Proc Natl Acad Sci U S A 113 E1710–1719, doi:10.1073/pnas.1522179113 (2016). 26951678 PMC4812758

[32] Yang J. The nucleotide pGpp acts as a third alarmone in Bacillus, with functions distinct from those of (p) ppGpp. Nat Commun 11, 5388, doi:10.1038/s41467-020-19166-1 (2020). 33097692 PMC7584652

[33] Irving S. E., Choudhury N. R. & Corrigan R. M. The stringent response and physiological roles of (pp)pGpp in bacteria. Nat Rev Microbiol, doi:10.1038/s41579-020-00470-y (2020). 33149273

[34] Diez S., Ryu J., Caban K., Gonzalez R. L. Jr. & Dworkin J. The alarmones (p)ppGpp directly regulate translation initiation during entry into quiescence. Proc Natl Acad Sci U S A 117, 15565–15572, doi:10.1073/pnas.1920013117 (2020). 32576694 PMC7354938

[35] Bittner A. N., Kriel A. & Wang J. D. Lowering GTP level increases survival of amino acid starvation but slows growth rate for Bacillus subtilis cells lacking (p)ppGpp. J Bacteriol 196, 2067–2076, doi:10.1128/jb.01471-14 (2014). 24682323 PMC4010990

[36] Tuomanen E., Cozens R., Tosch W., Zak O. & Tomasz A. The rate of killing of Escherichia coli by beta-lactam antibiotics is strictly proportional to the rate of bacterial growth. J Gen Microbiol 132, 1297–1304, doi:10.1099/00221287-132-5-1297 (1986). 3534137

[37] Kriel A. GTP dysregulation in Bacillus subtilis cells lacking (p)ppGpp results in phenotypic amino acid auxotrophy and failure to adapt to nutrient downshift and regulate biosynthesis genes. J Bacteriol 196, 189–201, doi:10.1128/jb.00918-13 (2014). 24163341 PMC3911124

[38] Handke L. D., Shivers R. P. & Sonenshein A. L. Interaction of Bacillus subtilis CodY with GTP. J Bacteriol 190, 798–806, doi:10.1128/JB.01115-07 (2008). 17993518 PMC2223590

[39] Lewis K. Multidrug tolerance of biofilms and persister cells. Curr Top Microbiol Immunol 322, 107–131, doi:10.1007/978-3-540-75418-3_6 (2008). 18453274

[40] Liu K. Molecular mechanism and evolution of guanylate kinase regulation by (p)ppGpp. Mol Cell 57, 735–749, doi:10.1016/j.molcel.2014.12.037 (2015). 25661490 PMC4336630

[41] Libby E. A., Reuveni S. & Dworkin J. Multisite phosphorylation drives phenotypic variation in (p)ppGpp synthetase-dependent antibiotic tolerance. Nat Commun 10, 5133, doi:10.1038/s41467-019-13127-z (2019). 31723135 PMC6853874

[42] D’Elia M. A. Probing teichoic acid genetics with bioactive molecules reveals new interactions among diverse processes in bacterial cell wall biogenesis. Chemistry and Biology 16, 548–556, doi:10.1016/j.chembiol.2009.04.009 (2009). 19477419

[43] Veening J. W., Smits W. K. & Kuipers O. P. Bistability, epigenetics, and bet-hedging in bacteria. Annu Rev Microbiol 62, 193–210, doi:10.1146/annurev.micro.62.081307.163002 (2008). 18537474

[44] Miller C. SOS response induction by beta-lactams and bacterial defense against antibiotic lethality. Science 305, 1629–1631, doi:10.1126/science.1101630 (2004). 15308764

[45] D’Elia M. A. Probing teichoic acid genetics with bioactive molecules reveals new interactions among diverse processes in bacterial cell wall biogenesis. Chem Biol 16, 548–556, doi:10.1016/j.chembiol.2009.04.009 (2009). 19477419

[46] Eiamphungporn W. & Helmann J. D. The Bacillus subtilis sigma(M) regulon and its contribution to cell envelope stress responses. Mol Microbiol 67, 830–848, doi:10.1111/j.1365-2958.2007.06090.x (2008). 18179421 PMC3025603

[47] Katz E. & Demain A. L. The peptide antibiotics of Bacillus: chemistry, biogenesis, and possible functions. Bacteriol Rev 41, 449–474 (1977). 70202 10.1128/br.41.2.449-474.1977 PMC414008

[48] Geiger T., Kastle B., Gratani F. L., Goerke C. & Wolz C. Two small (p)ppGpp synthases in Staphylococcus aureus mediate tolerance against cell envelope stress conditions. J Bacteriol 196, 894–902, doi:10.1128/JB.01201-13 (2014). 24336937 PMC3911181

[49] Lazar V., Snitser O., Barkan D. & Kishony R. Antibiotic combinations reduce Staphylococcus aureus clearance. Nature 610, 540–546, doi:10.1038/s41586-022-05260-5 (2022). 36198788 PMC9533972

[50] Martinez J. L. Environmental pollution by antibiotics and by antibiotic resistance determinants. Environ Pollut 157, 2893–2902, doi:10.1016/j.envpol.2009.05.051 (2009). 19560847

[51] Janes B. K. & Stibitz S. Routine markerless gene replacement in Bacillus anthracis. Infect Immun 74, 1949–1953, doi:10.1128/IAI.74.3.1949-1953.2006 (2006). 16495572 PMC1418658

[52] Koo B. M. Construction and Analysis of Two Genome-Scale Deletion Libraries for Bacillus subtilis. Cell Syst 4, 291–305 e297, doi:10.1016/j.cels.2016.12.013 (2017). 28189581 PMC5400513

[53] de Kok S. Rapid and reliable DNA assembly via ligase cycling reaction. ACS Synth Biol 3, 97–106, doi:10.1021/sb4001992 (2014). 24932563

[54] Griffith K. L. & Grossman A. D. Inducible protein degradation in Bacillus subtilis using heterologous peptide tags and adaptor proteins to target substrates to the protease ClpXP. Mol Microbiol 70, 1012–1025, doi:10.1111/j.1365-2958.2008.06467.x (2008). 18811726 PMC2581644

[55] Harwood C. R. & Cutting S. M. Molecular biological methods for Bacillus. (Wiley, 1990).

[56] Adimpong D. B. Antimicrobial susceptibility of Bacillus strains isolated from primary starters for African traditional bread production and characterization of the bacitracin operon and bacitracin biosynthesis. Appl Environ Microbiol 78, 7903–7914, doi:10.1128/AEM.00730-12 (2012). 22941078 PMC3485967

[57] Clasquin M. F., Melamud E. & Rabinowitz J. D. LC-MS data processing with MAVEN: a metabolomic analysis and visualization engine. Curr Protoc Bioinformatics Chapter 14, Unit14 11, doi:10.1002/0471250953.bi1411s37 (2012). PMC4055029 22389014

[58] Young J. W. Measuring single-cell gene expression dynamics in bacteria using fluorescence time-lapse microscopy. Nat Protoc 7, 80–88, doi:10.1038/nprot.2011.432 (2011). 22179594 PMC4161363

[59] Johnson C. M. & Grossman A. D. Identification of host genes that affect acquisition of an integrative and conjugative element in Bacillus subtilis. Mol Microbiol 93, 1284–1301, doi:10.1111/mmi.12736 (2014). 25069588 PMC4160349

[60] van Opijnen T., Lazinski D. W. & Camilli A. Genome-Wide Fitness and Genetic Interactions Determined by Tn-seq, a High-Throughput Massively Parallel Sequencing Method for Microorganisms. Curr Protoc Mol Biol 106, 7 16 11–24, doi:10.1002/0471142727.mb0716s106 (2014). PMC4568079 24733243

